# Frontal Underactivation During Working Memory Processing in Adults With Acute Partial Sleep Deprivation: A Near-Infrared Spectroscopy Study

**DOI:** 10.3389/fpsyg.2018.00742

**Published:** 2018-05-16

**Authors:** Michael K. Yeung, Tsz L. Lee, Winnie K. Cheung, Agnes S. Chan

**Affiliations:** ^1^Neuropsychology Laboratory, Department of Psychology, Chinese University of Hong Kong, Hong Kong, China; ^2^Chanwuyi Research Center for Neuropsychological Well-Being, Chinese University of Hong Kong, Hong Kong, China

**Keywords:** sleep deprivation, frontal lobe, working memory, *n*-back, hemodynamics, near-infrared spectroscopy

## Abstract

Individuals with partial sleep deprivation may have working memory (WM) impairment, but the underlying neural mechanism of this phenomenon is relatively unknown. The present study examined neural processing during WM performance in individuals with and without partial sleep deprivation using near-infrared spectroscopy (NIRS). Forty college students (10 males) were equally split into Sufficient Sleep (SS) and Insufficient Sleep (IS) groups based on self-reports of previous night's sleep duration. Participants in the SS group obtained the recommended amounts of sleep according to various sleep organizations (i.e., >7.0 h), whereas those in the IS group obtained amounts of sleep no greater than the lower limit of the recommendation (i.e., ≤7.0 h). All participants underwent an *n*-back paradigm with a WM load (i.e., 3-back) and a control condition (i.e., 0-back) while their prefrontal hemodynamics were recorded by NIRS. The IS and SS groups performed the tasks comparably well. However, unlike the SS group, which exhibited bilateral frontal activation indicated by increased oxyhemoglobin concentration and decreased deoxyhemoglobin concentration during WM processing (i.e., 3-back > 0-back), the IS group did not exhibit such activation. In addition, levels of WM-related frontal activation, especially those on the left side, correlated with sleep duration the night before, even when habitual sleep duration was controlled for. The findings suggest the presence of frontal lobe dysfunction in the absence of evident WM difficulties in individuals with acute partial sleep deprivation. They also highlight the importance of a good night's sleep to brain health.

## Introduction

A substantial number of studies have shown that partial sleep deprivation is very common in the daily life of adults in modern society (Lund et al., 2010; National Sleep Foundation, 2013; Vargas et al., 2014; Quick et al., 2016). That is, although numerous sleep organizations, including the National Sleep Foundation, American Academy of Sleep Medicine, and Sleep Research Society, recommend at least 7 h of sleep per night for adults aged between 18 and 64 years (Hirshkowitz et al., 2015; Watson et al., 2015), the International Bedroom Poll in 2013 revealed that 29–66% and 15–33% of young and middle-aged adults in six different countries, including the United States, Canada, Mexico, United Kingdom, Germany, and Japan, reported obtaining fewer than 7 h of sleep at night on weeknights and weekends, respectively (National Sleep Foundation, 2013). In addition, some studies have estimated that 25–41% of college students (i.e., a subgroup of adults) obtain less than the amount of sleep recommended by sleep organizations every night (Lund et al., 2010; Vargas et al., 2014; Quick et al., 2016). Given such a high prevalence of sleep restriction in the population, it is important to know whether individuals who do not obtain the recommended amounts of sleep (i.e., those who obtain insufficient sleep) exhibit cognitive and neural dysfunction compared with those who obtain the recommended amounts of sleep (i.e., sufficient sleep) at night.

Previous research has consistently shown that sleep deprivation negatively affects various domains of cognitive function (Lim and Dinges, 2010), including working memory (WM) (Frenda and Fenn, 2016). WM refers to the temporary maintenance and manipulation of information necessary for complex tasks (Baddeley and Hitch, 1974). Empirical studies have consistently found that compared with a well-rested control condition (e.g., 8 h of sleep at night), 1–2 nights of acute total sleep deprivation (i.e., a consecutive 24–48 h of wakefulness) could lead to behavioral impairments in verbal (Chee and Choo, 2004; Habeck et al., 2004; Choo et al., 2005; Mu et al., 2005; Turner et al., 2007; Lo et al., 2012; Lythe et al., 2012) and visual WM (Chee and Chuah, 2007; Drummond et al., 2012). In addition, some studies have suggested a negative and accumulative effect of partial sleep deprivation (i.e., sleep restriction to ≤ 7 h/night) on WM performance (Casement et al., 2006; Del Angel et al., 2015). That is, although 1 or 4 nights of sleep restriction to no more than 4 h per night might not be sufficient to lead to significant WM impairments (Miyata et al., 2010; Drummond et al., 2012), 5 to 6 consecutive nights of sleep restriction to 4 h per night have been consistently found to result in significant WM deficits (Lo et al., 2012, 2016; Del Angel et al., 2015). Based on these findings, it appears that individuals who experience sleep deprivation may exhibit WM deficits compared with those having sufficient sleep and that the severity of WM impairment increases with that of sleep deprivation.

In addition, a diverse array of studies has empirically examined the neural basis of WM, and they have suggested a critical involvement of the prefrontal cortex (PFC), particularly its lateral section, during WM performance. Specifically, human lesion studies have shown that damage to the PFC results in WM deficits (Müller et al., 2002; Tsuchida and Fellows, 2009). In addition, functional neuroimaging studies, such as those utilizing functional magnetic resonance imaging (fMRI; Braver et al., 1997; D'Esposito et al., 2000; Owen et al., 2005) and near-infrared spectroscopy (NIRS; Ehlis et al., 2008; Herff et al., 2013; Yeung et al., 2016), have shown that the PFC is activated in response to WM load irrespective of the sensory modality of the information. Thus, given that the PFC plays an essential role in WM and that many studies have reported a negative effect of sleep deprivation on WM performance, it is reasonable to speculate that sleep deprivation might lead to a dysfunction of the PFC during performance of WM tasks. To our knowledge, some fMRI studies have been conducted to investigate the effect of total sleep deprivation on neural processing during WM performance, and they have consistently reported a change in frontal activation levels during WM tasks following a complete loss of 1–2 nights of sleep (Habeck et al., 2004; Choo et al., 2005; Mu et al., 2005; Lythe et al., 2012). Specifically, these studies have found that compared with a well-rested condition in which participants obtained approximately 8 h of sleep on the previous night, consecutive 24-, 30-, 31-, or 48-h sleep deprivation would lead to a reduction in frontal activation specifically associated with WM processing (i.e., WM load > vigilance control) in adults.

Based on the previous findings of a potential negative effect of partial sleep deprivation on WM (Del Angel et al., 2015) and of a link between total sleep deprivation and frontal lobe dysfunction during WM tasks (Choo et al., 2005; Lythe et al., 2012), it is conceivable that partial sleep deprivation may also negatively affect frontal lobe processing during WM performance. However, to our knowledge, few studies have examined the association between partial sleep deprivation and the neural processing associated with WM performance. Therefore, it remains uncertain whether individuals who obtain restricted amounts of sleep at night would exhibit frontal lobe dysfunction during WM processing. Thus, to enhance our understanding of the relationship between partial sleep deprivation and neural processing during WM performance, the present study aimed to examine the neural processing that underlies WM processing in individuals with partial sleep deprivation.

Near-infrared spectroscopy (NIRS) is an optical imaging method that uses light in the near-infrared spectrum (650–950 nm) to non-invasively monitor the hemodynamic responses evoked by neuronal activity (Villringer and Chance, 1997). The technique measures quantitative changes in the concentrations of oxygenated hemoglobin ([oxy-Hb]) and deoxygenated hemoglobin ([deoxy-Hb]) in cerebral blood, which have been shown to significantly correlate with the blood oxygenation level-dependent (BOLD) signal as measured by fMRI (Strangman et al., 2002; Cui et al., 2011; Sato et al., 2013). In addition, cerebral activation has been shown to result in an increase in [oxy-Hb] and a decrease in [deoxy-Hb] (Hock et al., 1995). To our knowledge, many NIRS studies have adopted an *n*-back paradigm to examine neural processing during WM performance in both the healthy and clinical populations (Hoshi et al., 2003; Ehlis et al., 2008; Pu et al., 2011; Herff et al., 2013; Koike et al., 2013; Yeung et al., 2016), and they have consistently reported that WM processing (e.g., the 3-back > 0-back contrast) would specifically lead to frontal activation indicated by increases in [oxy-Hb], decreases in [deoxy-Hb], or both in healthy individuals (Hoshi et al., 2003; Herff et al., 2013; Koike et al., 2013); these findings are consistent with those reported in the fMRI literature (Owen et al., 2005). In addition, some NIRS studies have reported reduced WM-related frontal activation (e.g., reduced [oxy-Hb] increase on the 2-back > 0-back contrast) in a number of neuropathological populations, such as patients with schizophrenia (Koike et al., 2013), major depressive disorder (Pu et al., 2011), attention-deficit/hyperactive disorder (Ehlis et al., 2008), and mild cognitive impairment (Yeung et al., 2016), suggesting that NIRS could be used to reveal differences in frontal lobe processing among populations with different mental conditions. Taken together, previous studies have supported the use of NIRS to examine neural processing during WM tasks.

Thus, to enhance our understanding of the relationship between partial sleep deprivation and neural processing underlying WM performance and to examine the applicability of NIRS to sleep research, the present study utilized NIRS to examine neural processing during WM processing in individuals with partial sleep deprivation. We adopted a digit *n*-back paradigm with a WM load (i.e., 3-back) and a vigilance control condition (i.e., 0-back) to isolate the neural processes associated with WM processing (i.e., the 3-back vs. 0-back contrast). Based on the existing fMRI literature on total sleep deprivation (Choo et al., 2005; Lythe et al., 2012) and on the existing NIRS literature on WM (Ehlis et al., 2008; Yeung et al., 2016), we hypothesized that compared to individuals with sufficient sleep, those with insufficient sleep would exhibit reduced frontal activation indicated by smaller [oxy-Hb] increases and [deoxy-Hb] decreases during WM processing.

## Methods

### Participants

A total of 44 right-handed college students aged between 18 and 24 years (11 males, 33 females) were initially recruited from the subject pool of the Department of Psychology at the Chinese University of Hong Kong in exchange for course credits for their participation. Four participants were subsequently excluded from this study because of missing data due to technical issues (*n* = 2), failure to perform the *n*-back task (i.e., 0 correct hits for the first task session; *n* = 1), or being on medication (*n* = 1). Thus, the final sample consisted of 40 participants (10 males, 30 females). None of the participants reported alcohol intake or drug use on the day of the experiment. None reported a history of any neurological or psychiatric disorders. All of them had normal or corrected-to-normal vision.

To compare neural processing during WM performance between individuals with and without partial sleep deprivation, the whole sample was split retrospectively into Insufficient Sleep (IS; *n* = 20) and Sufficient Sleep (SS; *n* = 20) groups based on self-reports of sleep duration on the previous night. According to the nightly sleep durations recommended for adults by various sleep organizations, such as the National Sleep Foundation, American Academy of Sleep Medicine, and Sleep Research Society (i.e., 7–9 h of sleep/night; Hirshkowitz et al., 2015; Watson et al., 2015), each participant in the IS group had a sleep duration no greater than the lower limit of the recommended sleep durations (i.e., ≤ 7.0 h) the night before. In addition, each participant in the SS group had a sleep duration within the range of the recommended sleep durations (i.e., >7.0 h) the night before. In addition, the cut-off of 7 h was chosen because research studies have shown that sleep restricted to ≤ 7.0 h per night could lead to cognitive impairments in the long run (Belenky et al., 2003). Some prior research has shown significant moderate-to-large correlations between self-reported and objectively measured sleep duration (i.e., rs from 0.43 to 0.45; Lauderdale et al., 2008; Cespedes et al., 2016), suggesting that self-reports convey important information about sleep patterns.

It should be noted that individuals who reported obtaining 7.0 h of sleep the night before were assigned to the IS group because consecutive nights of 7.0 h of sleep per night have been shown to result in a mild decline in psychomotor speed (Belenky et al., 2003). In addition, the results did not significantly change after we excluded these individuals from the analyses. Moreover, we did not split the sample by habitual sleep duration for several reasons: (1) individuals who obtained insufficient sleep habitually also obtained significantly less sleep during the previous night than those who habitually obtained sufficient sleep (i.e., >7.0 h/night on average over the past month), *p* = 0.020; (2) sleep duration on the previous night, or a single night's sleep duration, was significantly correlated with habitual sleep duration, *r*_(40)_ = 0.31, *p* = 0.049; thus, the two sleep durations might reflect a shared sleep phenomenon; and (3) levels of WM-related frontal activation were uniquely associated with previous night's sleep duration rather than habitual sleep duration (see the last paragraph of the Results section).

The experiment was conducted either in the morning or afternoon on a college weekday (i.e., starting somewhere between 09:30 and 12:00 or between 14:30 and 16:30). Thus, most of the participants in the IS group were partially deprived of sleep at week nights. The SS and IS groups did not significantly differ in time of experiment (in 24-h clock; SS: *M* = 12:42, *SD* = 01:59; IS: *M* = 13:29, *SD* = 02:33), *t*_(38)_ = 1.07, *p* = 0.29, or in the interval between wake-up time and time of experiment, *p* = 0.81. More importantly, as shown in section Exploratory Analyses on the Effects of Time on Task and Time of Experiment, time of experiment did not confound any of the present behavioral or NIRS findings.

### Procedure and materials

The entire experiment, which lasted approximately 45 min, consisted of two parts. First, the participants were asked to complete a questionnaire on their demographic and academic background and the amounts of sleep they obtained during the previous night and habitually (i.e., the average nightly sleep duration over the past 1 month, weekdays, and weekends combined). Second, the participants underwent the *n*-back paradigm during NIRS recording. All participants provided written informed consent in accordance with the Declaration of Helsinki prior to the experiment. The study protocol was approved by the Joint Chinese University of Hong Kong–New Territories East Cluster Clinical Research Ethics Committee.

A digit *n*-back paradigm adapted from previous NIRS studies was employed as the main task (Ehlis et al., 2008; Yeung et al., 2016). The task consisted of a WM load condition (i.e., 3-back) and a vigilance control condition (i.e., 0-back). All participants underwent this paradigm while their prefrontal hemodynamics were recorded by NIRS. In this paradigm, each participant completed two *n*-back runs, which were separated by a break. During each run, the 0- and 3-back tasks were repeated twice and the two tasks were performed alternately in blocks (i.e., either a 0-3-0-3 or 3-0-3-0 sequence). The order of the 0- and 3-back blocks was counterbalanced both within and across participants to avoid order effects. That is, half of the participants performed the 3-0-3-0 sequence the first time and the 0-3-0-3 sequence the second time, whereas others performed the 0-3-0-3 sequence the first time and the 3-0-3-0 sequence the second time.

**Figure 1:**
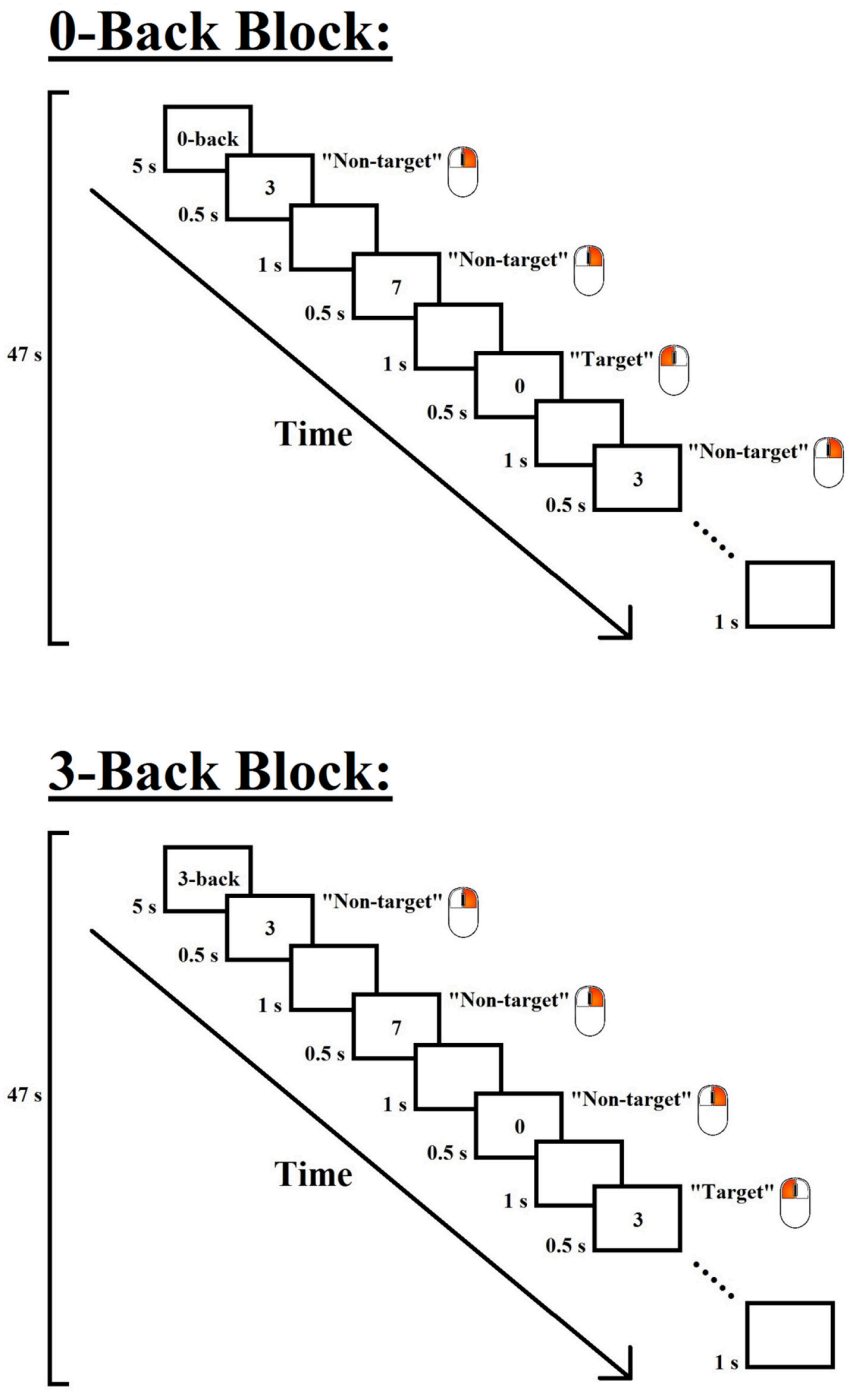
Experimental design of the 0- and 3-back conditions of the *n*-back paradigm. Each task block began with a 5-s instruction cue, followed by 28 trials (7 target and 21 non-target trials) that were presented in a pseudo-randomized order. In each trial, a digit was displayed for 500 ms, followed by an inter-stimulus interval of 1,000 ms. Participants were required to press the left button of a mouse when the target stimulus appeared but to press the right button for the other stimuli. The target stimulus for the 0-back condition was the digit “0”, and that for the 3-back condition was the digit that was identical to the one presented three trials before.

The design of each task block of the 0- and 3-back conditions is shown in Figure 1. Each task block consisted of 28 trials, including 7 target and 21 non-target trials that were presented in a pseudo-randomized order. Each trial consisted of a digit, which was presented in the center of the screen for 500 ms, followed by an inter-stimulus interval of 1,000 ms. In the 0-back condition, the participants were required to press the left button of a mouse with their right index finger when the digit “0” (i.e., target) appeared but to press the right button with their right middle finger for all other stimuli (i.e., non-targets); in the 3-back condition, the participants were required to press the left button when the presented digit was identical to the one presented three trials before (i.e., target) but to press the right button for all other stimuli (i.e., non-targets). To minimize task confusion and maximize task engagement, each task block began with a 5-s cue that indicated the following task, and the task blocks were interleaved with 30-s rest periods. Each task block lasted 47 s, and each *n*-back session lasted approximately 5.5 min.

Before the experiment actually began, the participants were first briefed on the task instructions and procedure. Specifically, they were instructed to perform the 0- and 3-back tasks alternately several times and were informed that there would be a 30-s rest after each task block. Each of them then practiced the 0- and 3-back tasks to become familiar with them. All stimuli were presented using E-Prime 1.2 software (Psychology Software Tools, Pittsburgh, PA).

### NIRS measurement

**Figure 2:**
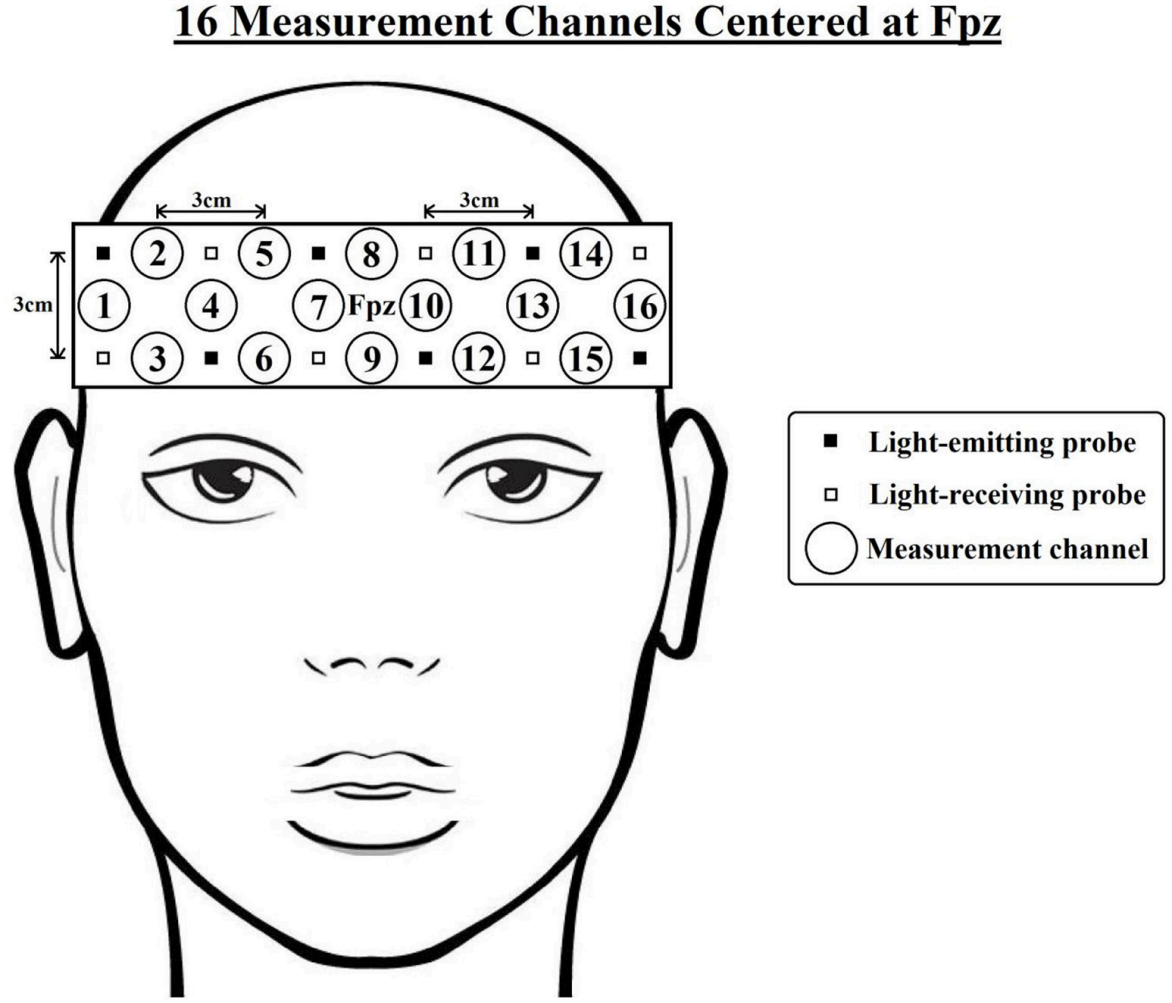
Arrangement of the near-infrared spectroscopy (NIRS) channels. The distance between each pair of emitter and detector probes is 3 cm. In accordance with the international 10/20 system, the center of the probe matrix was placed on Fpz, and the probes at the bottom left and right corners were placed around F7 and F8, respectively. Based on previous studies (Okamoto et al., 2004; Koessler et al., 2009), it was estimated that the outermost channels (e.g., ch1-4,13-16) were located around the lateral prefrontal cortices, and the medial channels (e.g., ch7-10) were located around the medial frontopolar cortex and dorsomedial prefrontal cortex.

Changes in absorbed near-infrared light were recorded using a 16-channel OEG-SpO2 system (Spectratech Inc., Tokyo, Japan). The machine uses two wavelengths of near-infrared light, 770 and 840 nm, and calculates the relative [oxy-Hb] and [deoxy-Hb] changes based on the modified Beer-Lambert Law (Delpy et al., 1988); the sampling rate is 12.21 Hz. The sensor consists of 6 emission and 6 detector probes arranged in a 2-row × 6-column matrix, which was centered on the participant's forehead (see Figure 2). The distance between each pair of emitter and detector probes was 3 cm, thus measuring [oxy-Hb] and [deoxy-Hb] changes at a depth of 2–3 cm below the scalp (Toronov et al., 2001). NIRS data were collected at 16 measurement channels located between each pair of emitter and detector probes. In accordance with the international 10/20 system (Jasper, 1958), the center of the probe matrix was placed on Fpz, and the probes at the bottom left and right corners were placed around F7 and F8, respectively. Based on previous studies that measured the anatomical locations of international 10–20 cortical projection points (Okamoto et al., 2004; Koessler et al., 2009), it was estimated that the outermost channels (e.g., ch1-4,13-16) were located around the lateral prefrontal cortices, and the medial channels (e.g., ch7-10) were located around the medial frontopolar cortex and dorsomedial prefrontal cortex.

### Data analysis

For the continuous and categorical data variables, the IS and SS groups were compared using independent sample *t*-tests and chi-squared tests, respectively. For the 0- and 3-back tasks, we calculated the mean reaction time (RT) separately for correct hits and correct rejections. Trial outliers, defined as RTs that were shorter than 150 ms or 2.5 *SD*s above each individual's mean, were excluded from the calculation of the mean RT for each individual. In addition, for each *n*-back condition, we calculated the non-parametric index of sensitivity *A*′ (Snodgrass and Corwin, 1988), which is a performance index that considers both the hit rate (*H*) and the false alarm rate (*FA*). The formula for calculating this index is [0.5 + [(*H* – *FA*)(1 + *H* – *FA*)]/[4*H*(1 – *FA*)] for *H* ≥ *FA* and [0.5 – [(*FA* – *H*)(1 + *FA* – *H*)]/[4*FA*(1 – *H*)] for *FA* > *H. A*′ ranges from 0 to 1, and a larger *A*′ implies better discriminability or more accurate performance. Because the current *n*-back paradigm involves unequal numbers of target and non-target trials in a ratio of 1:3, *A*′ was used instead of accuracy because it is less affected by response biases. That is, accuracy is 25% if a participant responds with “target” in all trials and 75% if the participant responds with “non-target” in all trials, whereas *A*′ would remain the same (i.e., 0.5) and reflect chance-level performance in both cases.

For the NIRS data, a 0.10-Hz low-pass filter at a slope of 60 dB/oct was first applied to remove short-term motion and cardiac artifacts. Then, to correct for the slow drift of signals over time, we performed a first-order linear fitting for each task block based on the values of the pre- and post-task periods, as defined by the 10 s preceding the task block and the last 10 s of the following rest period, respectively. The slow-drift-corrected data were then averaged across time points (excluding the initial 5-s cue period), repetitions, and sessions to generate the mean [oxy-Hb] and [deoxy-Hb] levels for each *n*-back condition, channel, and participant. Slow-drift correction and averaging were performed using Matlab® R2014a (MathWorks, Natick, MA). To investigate WM-related frontal activation (i.e., the 3-back > 0-back contrast), paired *t*-tests were first performed to examine the differences in mean [oxy-Hb] and [deoxy-Hb] levels between the 3-back (i.e., WM task) and 0-back (i.e., control task) conditions for each channel within the IS and SS groups separately. Then, independent-sample *t*-tests were performed to compare the mean WM-related [oxy-Hb] changes (i.e., 3-back minus 0-back) between the IS and SS groups.

In addition, to describe the relationships between sleep duration, mean WM-related [oxy-Hb] and [deoxy-Hb] changes, and task performance variables, we calculated Pearson's correlations (two-tailed) for the whole sample. We did not perform correlation analyses for the IS and SS groups separately because of a limited range of the sleep duration variables in each group. For the NIRS data, we made corrections for multiple comparisons using the false discovery rate (FDR): we set the value of q^*^ and specified the maximum FDR as 0.05 so that there were no more than 5% false positives on average (Benjamini and Hochberg, 1995). However, the results with *p*s < 0.05 that nevertheless failed to survive the FDR correction were also reported because these findings might warrant follow-up in future studies. Raw *p*-values are reported in all cases. All of the statistical analyses were performed using SPSS 22.0 software (IBM Corporation, Armonk, NY, USA). The significance level was set to 0.05 for all tests.

## Results

### Demographic and sleep characteristics of the IS and SS groups

The demographic and sleep characteristics of the IS and SS groups are shown in Table 1. For the demographic variables, independent-sample *t*-tests or chi-squared tests showed that the two groups did not significantly differ in age, gender, or GPA, *p*s > 0.35. For the sleep variables, there was a significant group difference in sleep duration the night before the experimental session, *t*_(38)_ = 8.16, *p* < 0.001, with the IS group obtaining significantly less sleep the night before. In addition, there was a significant group difference in the sleep onset time the night before, *t*_(38)_ = 3.31, *p* = 0.002, but not in the wake time on the experimental day, *t*_(38)_ = 1.11, *p* = 0.27, suggesting that the group difference in sleep duration the night before was mainly attributed to a delayed sleep onset time in the IS group. Nevertheless, there were no significant group differences in habitual sleep duration, habitual sleep onset time, or habitual wake time, *p*s > 0.05. In addition, the sleep duration the night before was significantly shorter than the habitual sleep duration in the IS group, *t*_(19)_ = 5.30, *p* < 0.001, but the two sleep durations were statistically comparable in the SS group, *t*_(19)_ = −1.34, *p* = 0.20. Hence, the IS group could be characterized as having acute partial sleep deprivation.

**Table 1:** Demographic Characteristics and *n*-Back Task Performance of the Sufficient Sleep (SS; *n* = 20) and Insufficient Sleep (IS; *n* = 20) Groups.

	**Group**		
	**SS (*n* = 20)**	**IS (*n* = 20)**	***t*/* χ^2^***	***p***
Age (year)	20.02 (1.56)	19.60 (1.29)	0.94	0.35
Gender (Male/Female)	4/16	6/14	0.53	0.47
GPA (unit)	3.22 (0.31)	3.16 (0.26)	0.64	0.53
**SLEEP DURING THE PREVIOUS NIGHT**				
Sleep duration (h)	8.25 (0.70)	6.19 (0.89)	8.16	<0.001^***^
Sleep onset time (hh:mm)	00:04 (01:33)	02:12 (01:22)	3.31	0.002^**^
Wake time (hh:mm)	09:02 (01:40)	08:29 (01:26)	1.11	0.27
**SLEEP OVER THE PAST 1 MONTH**				
Habitual sleep duration (h)	7.93 (1.19)	7.65 (1.17)	0.75	0.46
Habitual sleep onset time (hh:mm)	00:53 (01:06)	01:38 (01:15)	1.98	0.055
Habitual wake time (hh:mm)	08:49 (01:18)	09:17 (01:22)	1.09	0.28
**0-BACK**				
Mean correct rejection RT (ms)	322.36 (42.48)	324.23 (33.27)	0.16	0.88
Mean hit RT (ms)	389.42 (39.93)	389.81 (32.60)	0.03	0.97
*A*′ (unit)	0.98 (0.02)	0.99 (0.01)	1.11	0.28
**3-BACK**				
Mean correct rejection RT (ms)	468.72 (132.81)	429.89 (68.49)	1.16	0.26
Mean hit RT (ms)	552.00 (145.52)	517.11 (98.20)	0.89	0.38
*A*′ (unit)	0.86 (0.08)	0.89 (0.08)	1.16	0.25
**3-BACK MINUS 0-BACK**				
Mean correct rejection RT (ms)	146.36 (99.21)	105.66 (67.94)	1.51	0.14
Mean hit RT (ms)	162.58 (121.07)	127.31 (98.62)	1.01	0.32
*A*′ (unit)	−12.26 (7.42)	−9.89 (7.87)	0.98	0.33

*GPA, Grade Point Average. A's are the non-parametric indices of sensitivity on the n-back tasks. Sleep onset and wake times are reported in 24-h clock format [e.g., 00:04 (01:33) = 0:04 a.m. (1 h 33 min)]. Standard deviations are in parentheses*.

** *p < 0.01*;

*** *p < 0.001*.

### *n*-back task performance in the IS and SS groups

For the *n*-back task performance (see Table 1), mixed-design ANOVAs with Condition (0-back, 3-back) as the within-subjects factor and Group (SS, IS) as the between-subjects factor were conducted on *A*′ and mean RT separately. For all variables, the ANOVA results showed that the main effect of condition was significant, *p*s < 0.001, and neither the main effect of group nor the interaction between group and condition was significant, *p*s > 0.14. In addition, we carried out paired and independent-sample *t*-tests to compare the effects of WM load within and between groups, respectively. Paired *t*-tests conducted on the 0- and 3-back mean RTs and *A*′s within the IS and SS groups separately showed that both groups performed significantly more slowly and less accurately on the 3-back task than on the 0-back task, *p*s < 0.001. In addition, even when multiple comparisons were not corrected, independent-sample *t*-tests showed that the two groups did not significantly differ in any of the task performance indices, including the 0-back and 3-back *A*′s and mean RTs and the condition differences in *A*′ and mean RT, *p*s > 0.14. Thus, the results suggest that the groups performed comparably well on the tasks. None of the task performance variables significantly correlated with sleep duration the night before, *p*s > 0.13.

### WM-related frontal activation within and between the IS and SS groups

**Figure 3:**
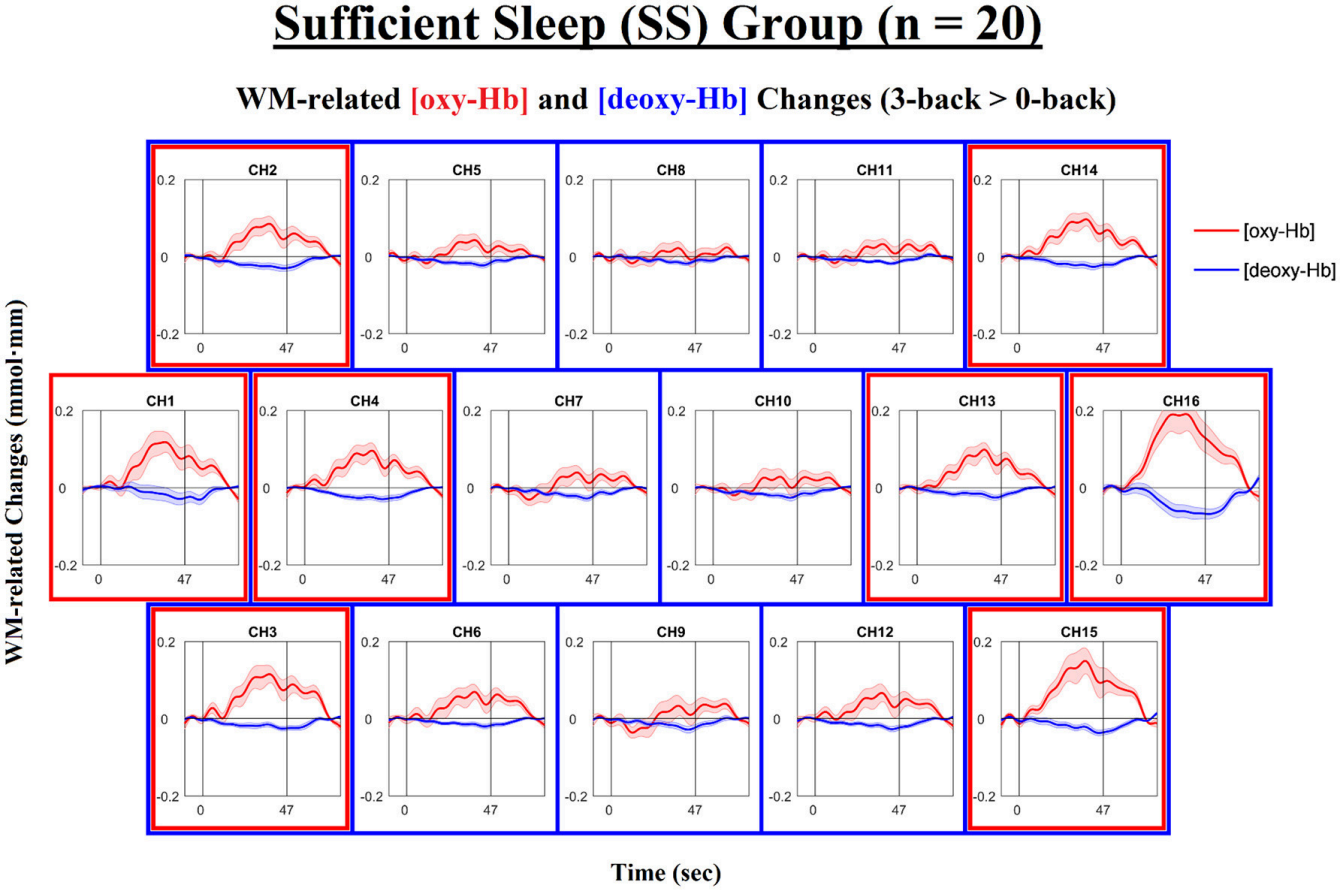
Time courses of the working-memory-related (WM-related, i.e., 3-back minus 0-back) [oxy-Hb] and [deoxy-Hb] changes in the Sufficient Sleep group (SS; *n* = 20). Each *n*-back block started at 0 s, began with an instruction cue that lasted for 5 s, and ended at 47 s. Channels that exhibited significant WM-related [oxy-Hb] increases (i.e., mean [oxy-Hb] values over the 42-s active task period, excluding the cue period) and those that exhibited significant WM-related [deoxy-Hb] decreases (i.e., mean [deoxy-Hb] values over the 42-s active task period, excluding the cue period) are denoted by red (ch1-4,13-16; *p*s < 0.05; FDR-corrected) and blue square borders (ch2-16; *p*s < 0.05; FDR-corrected), respectively. Lines and shading indicate the mean ± 1 standard error.

**Figure 4:**
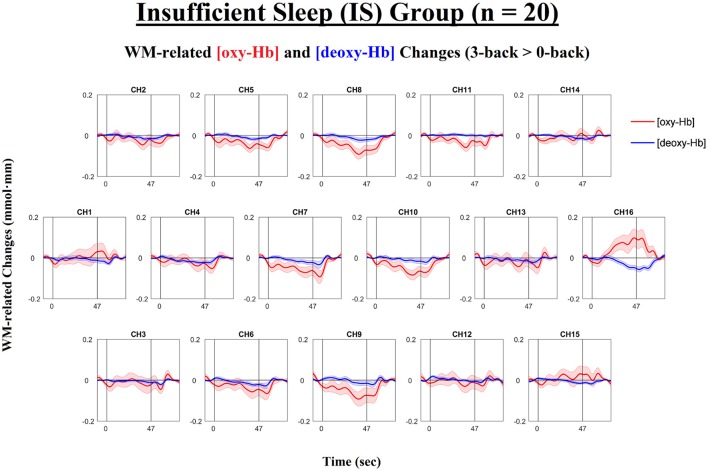
Time courses of the working-memory-related (WM-related, i.e., 3-back minus 0-back) [oxy-Hb] and [deoxy-Hb] changes in the Insufficient Sleep group (IS; *n* = 20). Each *n*-back block started at 0 s, began with an instruction cue that lasted for 5 s, and ended at 47 s. None of the channels exhibited significant WM-related [oxy-Hb] increases (i.e., mean [oxy-Hb] values over the 42-s active task period, excluding the cue period) or significant WM-related [deoxy-Hb] decreases (i.e., mean [deoxy-Hb] values over the 42-s active task period, excluding the cue period) (*p*s < 0.05; FDR-corrected). Lines and shading indicate the mean ± 1 standard error.

**Figure 5:**
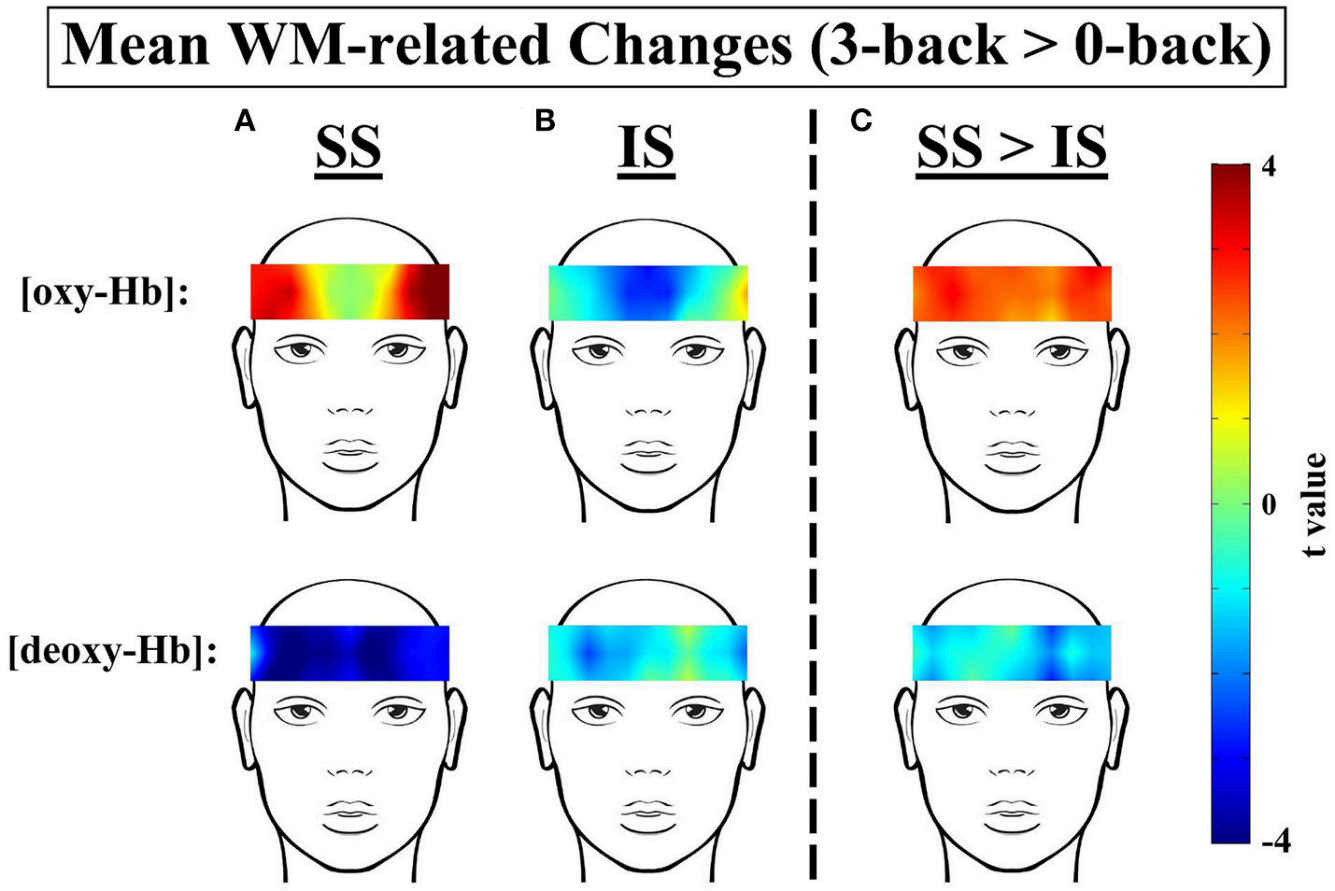
*t*-Contrast maps of the mean working-memory-related (WM-related, i.e., 3-back > 0-back) [oxy-Hb] and [deoxy-Hb] changes in the **(A)** Sufficient Sleep (SS; *n* = 20) and **(B)** Insufficient Sleep (IS; *n* = 20) groups and **(C)** between the SS and IS groups. For the top panels of **(A,B)**, red and blue indicate WM-related [oxy-Hb] increases and decreases, respectively; for the bottom panels of **(A,B)**, red and blue indicate WM-related [deoxy-Hb] increases and decreases, respectively. In addition, for the top panel of **(C)**, red indicates greater WM-related [oxy-Hb] increases in the SS group; for the bottom panel of **(C)**, blue indicates greater WM-related [deoxy-Hb] decreases in the SS group.

Next, we examined the levels of WM-related frontal activation (i.e., 3-back > 0-back) as measured by NIRS in the IS and SS groups separately. The time courses of the mean WM-related changes in [oxy-Hb] and [deoxy-Hb] are shown in Figure 3 for the SS group and in Figure 4 for the IS group. In addition, the topographical distributions of these changes are shown in Figure 5A for the SS group and in Figure 5B for the IS group. First, mixed-design ANOVAs with Condition (0-back, 3-back) and Channel (ch1-16) as the within-subjects factors and Group (SS, IS) as the between-subjects factor were conducted on mean [oxy-Hb] and [deoxy-Hb] levels separately. The ANOVA results showed a (marginally) significant group-by-condition interaction for both [oxy-Hb], *F*_(1, 38)_ = 7.29, *p* = 0.010, $\eta_{\text{p}}^{2}$ = 0.16, and [deoxy-Hb] variables, *F*_(1, 38)_ = 3.50, *p* = 0.069, $\eta_{\text{p}}^{2}$ = 0.057, suggesting group differences in WM-related [oxy-Hb] and [deoxy-Hb] changes. Thus, we carried out *t*-tests for each individual channel to clarify the spatial distribution of frontal hemodynamic changes within groups and to compare it between groups; FDR correction was applied to avoid inflation of Type I errors.

For the SS group (see Figures 3, 5A), the results of the paired *t*-tests showed that there were significant mean WM-related [oxy-Hb] increases on 8 out of 16 channels, all of which are located in the lateral PFC regions on both sides (ch1-4,13-16; FDR-corrected; *t*s from 2.75 to 4.45, *p*s from 0.00028 to 0.013, *d*s from 0.78 to 0.99). In addition, there were significant mean WM-related [deoxy-Hb] decreases on all but one channel (ch2-16; FDR-corrected; *t*s from 2.63 to 4.53, *p*s from 0.00023 to 0.017, *d*s from 0.59 to 1.01). In contrast, for the IS group (see Figures 4, 5B), the results of the paired *t*-tests showed that there were no significant mean WM-related [oxy-Hb] or [deoxy-Hb] changes on any of the 16 channels (FDR-corrected; *p*s > 0.003), although marginally significant mean [oxy-Hb] (ch7-10; uncorrected; *t*s from −2.96 to −2.16, *p*s from 0.008 to 0.044) or [deoxy-Hb] changes (ch4,16; uncorrected; *t*s from 2.31 to 2.52, *p*s from 0.021 to 0.032) were found on some channels.

Most importantly, there were group differences in the levels of WM-related frontal activation (see Figure 5C). Specifically, for the mean WM-related [oxy-Hb] changes, we found significant group differences on 10 bilateral channels (ch2-8,13-15; FDR-corrected; *t*s from 2.24 to 3.21, *p*s from 0.003 to 0.031, *d*s from 0.71 to 1.04) and marginally significant group differences on 2 left channels (ch10,16; uncorrected; *t*s from 2.16 to 2.19, *p*s from 0.035 to 0.038, *d*s from 0.68 to 0.69), such that the SS group had greater WM-related [oxy-Hb] increases in the bilateral PFC than the IS group. In addition, for the mean WM-related [deoxy-Hb] changes, we found marginally significant group differences on 2 channels on the left side (ch11-12; uncorrected; *t*s from 2.60 to 2.83, *p*s from 0.007 to 0.01, *d*s from 0.84 to 0.92), such that the SS group had greater WM-related [deoxy-Hb] decreases in the left PFC compared to the IS group. Thus, when the [oxy-Hb] and [deoxy-Hb] data are taken together, the NIRS results suggest that a lack of sleep was associated with frontal underactivation in the bilateral PFC, particularly on the left side.

Neither the left nor right mean WM-related [oxy-Hb] or [deoxy-Hb] changes significantly correlated with task performance variables, including the 0-back and 3-back *A*′s and mean RTs and the condition differences in *A*′ and mean RT, *p*s > 0.20. In addition, there were no significant differences in mean WM-related [oxy-Hb] or [deoxy-Hb] changes, regardless of side, between high and low performers in the whole sample or within the SS and IS groups separately after a median split on 3-back *A*′, *p*s > 0.16. Thus, acute sleep restriction seems to differentially affect frontal lobe functioning.

### Relationship between sleep duration on the previous night and WM-related frontal activation on the day of the experiment

To examine the relationships between sleep duration on the previous night and the mean WM-related [oxy-Hb] and [deoxy-Hb] on the day of the experiment, we calculated Pearson's correlation for the whole sample. Because WM processing has been shown to lead to bilateral frontal activation (Owen et al., 2005), we first conducted correlation analyses for the left (ch10-16 combined) and right (ch1-7 combined) hemispheres. The results showed that the mean WM-related [oxy-Hb] changes on both the left side, *r*_(40)_ = 0.53, *p* < 0.001, and the right side, *r*_(40)_ = 0.57, *p* < 0.001, were significantly correlated with the number of hours of sleep the night before. In addition, the mean WM-related [deoxy-Hb] changes on the left side, *r*_(40)_ = −0.43, *p* = 0.006, but not the right side, *r*_(40)_ = −0.23, *p* = 0.15, were also significantly correlated with the number of hours of sleep the night before. None of the task performance indices were significantly correlated with any of the NIRS variables or with the duration of sleep the night before, *p*s > 0.13.

**Figure 6:**
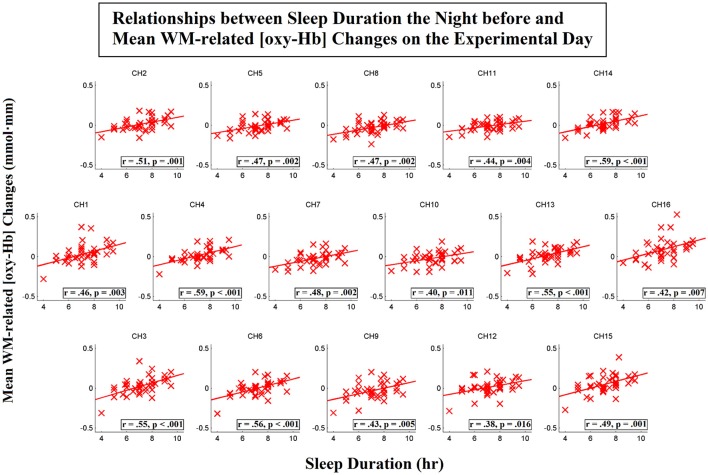
Correlations between sleep duration the night before and mean working-memory-related (WM-related, i.e., 3-back > 0-back) [oxy-Hb] changes on the experimental day. Pearson's correlation coefficient (*r*) was calculated for each channel, and a linear regression line was fitted to each channel. Channels that exhibited significant correlations are denoted by square borders (ch1-16; FDR-corrected; *p*s < 0.05; two-tailed).

**Figure 7:**
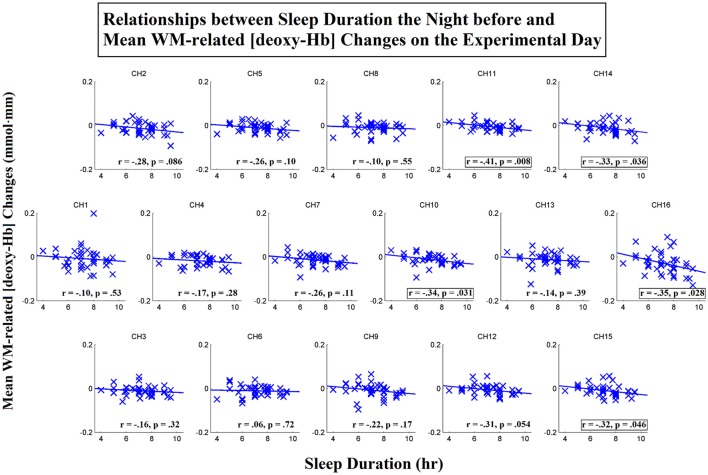
Correlations between sleep duration the night before and mean working-memory-related (WM-related, i.e., 3-back > 0-back) [deoxy-Hb] changes on the experimental day. Pearson's correlation coefficient (*r*) was calculated for each channel, and a linear regression line was fitted to each channel. Channels that exhibited marginally significant correlations are denoted by square borders (ch10-11,14-16; uncorrected; *p*s < 0.05; two-tailed).

To further investigate the spatial distribution of the correlations between the sleep duration the night before and the WM-related frontal activation on the day of the experiment, we then conducted correlation analyses for each individual channel. Scatterplots that represent these relationships for the [oxy-Hb] and [deoxy-Hb] variables are shown in Figures 6, 7, respectively. The results showed that the sleep duration the night before was significantly correlated with the mean WM-related [oxy-Hb] increases for all 16 channels (ch1-16; FDR-corrected; *r*s from 0.38 to 0.59, *p*s from 0.000053 to 0.016). In addition, we found marginally significant correlations between sleep duration the night before and the mean WM-related [deoxy-Hb] decreases for 5 channels on the left (ch10-11,14-16; uncorrected; *r*s from −0.32 to −0.41, *p*s from 0.008 to 0.046). Altogether, these results suggest that insufficient sleep the night before was associated with a lack of frontal activation during WM performance.

Finally, to examine the unique contribution of sleep duration the night before to the levels of WM-related frontal activation, we performed partial correlation analyses. The results showed that when habitual sleep duration was controlled for, sleep duration the night before remained significantly correlated with the mean WM-related [oxy-Hb] increases on both the left, *r*_(37)_ = 0.47, *p* = 0.003, and right sides, *r*_(37)_ = 0.54, *p* < 0.001. Specifically, significant correlations were found for 15 out of 16 channels (ch1-11,13-15; FDR-corrected; *r*s from 0.35 to 0.55, *p*s from 0.0004 to 0.031). In addition, sleep duration the night before remained significantly correlated with the mean WM-related [deoxy-Hb] decreases on the left side, *r*_(37)_ = −0.44, *p* = 0.005, but not the right side, *r*_(37)_ = −0.27, *p* = 0.10. Specifically, marginally significant correlations were found for 6 channels on the left side (ch10-12,14-16; uncorrected; *r*s from −0.32 to −0.43, *p*s from 0.007 to 0.046). Thus, these results suggest that the WM-related frontal underactivation was primarily attributed to insufficient sleep during the previous night (i.e., the effect of acute partial sleep deprivation).

### Exploratory analyses on the effects of time on task and time of experiment

We performed exploratory analyses to examine the effects of time on task by comparing the first and second halves of performance within each *n*-back series (e.g., the first 0-3 vs. the second 0-3 in the 0-3-0-3 series). First, mixed-design ANOVAs with Time (1st half, 2nd half) and Condition (0-back, 3-back) as the within-subjects factors and Group (IS, SS) as the between-subjects factor were conducted on *A*′ and mean RT separately. For both variables, the main effect of time and the interaction between condition and time were significant, *p*s < 0.015, but none of the main or interaction effects related to group was significant, *p*s > 0.14. Thus, there were no group differences in the time-on-task effects on performance. Then, mixed-design ANOVAs with Time (1st half, 2nd half) and Channel (ch1-16) as the within-subjects factors and Group (IS, SS) as the between-subjects factor were conducted on mean WM-related [oxy-Hb] and [deoxy-Hb] changes separately. For both variables, neither the main effect of time nor the interaction effects related to group was significant, *p*s > 0.18. Thus, there were no time-on-task effects on frontal hemodynamic changes or group differences in such effects.

To rule out the possibility that time of experiment confounded the findings, we conducted Pearson's correlation analyses (two-tailed; uncorrected) to determine the relationships between time of experiment and performance/NIRS variables in the whole sample. The results showed that time of experiment did not significantly correlate with any of the performance indices (i.e., 0-back and 3-back *A*′s and mean RTs and the condition differences in *A*′ and mean RT) or with any of the mean WM-related [oxy-Hb] or [deoxy-Hb] changes, regardless of side, *p*s > 0.09. In addition, the interval between wake-up time and time of experiment did not significantly correlate with any of the performance or NIRS variables either, *p*s > 0.19. Thus, time of experiment (and sleep inertia) did not seem to confound any of the performance or NIRS findings.

## Discussion

The primary objective of the present study was to utilize NIRS, an optical imaging technique, to examine neural processing during WM performance in individuals with partial sleep deprivation. Specifically, we examined WM-related frontal activation (i.e., 3-back > 0-back) in individuals who obtained amounts of sleep no greater than the lower limit of the sleep durations recommended by sleep organizations (i.e., the IS group; ≤ 7.0 h of sleep) and compared it with corresponding measurements from those who obtained the recommended amount of sleep on the previous night (i.e., the SS group; >7.0 h of sleep). The results showed that although the participants in the IS group performed the WM task as well as did those in the SS group, they exhibited an altered frontal activation pattern while performing the task. That is, unlike the participants in the SS group, who exhibited WM-related activation indicated by both [oxy-Hb] increases and [deoxy-Hb] decreases in the bilateral PFC, they did not exhibit such frontal activation. In addition, sleep duration the night before was found to positively correlate with the levels of WM-related frontal activation, particularly those in the left hemisphere, even after partialling out the effect of habitual sleep duration (i.e., average sleep duration over the past 1 month). Thus, the lack of frontal activation in response to WM load appears to be primarily attributable to sleep restriction over the previous night.

Regarding the behavioral results, we found that the participants in both the IS and SS groups performed significantly more slowly and less accurately on the 3-back condition than on the 0-back condition. This finding suggests that the presence of WM load resulted in a decrement in task performance and that our task manipulation (i.e., WM load vs. vigilance control) was successful. In addition, our finding of comparable task performance between individuals with sufficient sleep and those with insufficient sleep the previous night is consistent with the prior account of an accumulative effect of partial sleep deprivation on WM functioning. That is, 1 or 4 nights of acute partial sleep deprivation might not be sufficient to result in verbal WM impairments (Miyata et al., 2010; Drummond et al., 2012; Del Angel et al., 2015), yet 5 or more consecutive nights of sleep restriction would result in significant WM deficits (Lo et al., 2012, 2016; Del Angel et al., 2015). Thus, the absence of significant WM deficits in the IS group may be attributable to the presence of only mild sleep deprivation in this group of individuals.

Regarding the NIRS results, we found that individuals with sufficient sleep the night before exhibited bilateral frontal activations indicated by both [oxy-Hb] increases and [deoxy-Hb] decreases in response to WM load. These neural activation patterns are consistent with those reported by previous fMRI and NIRS studies also adopting the *n*-back paradigm (Braver et al., 1997; D'Esposito et al., 2000; Owen et al., 2005; Ehlis et al., 2008; Herff et al., 2013; Yeung et al., 2016). In addition, we found that these WM-related activations were spread extensively across the lateral prefrontal and frontopolar regions. Previous studies have shown the engagement of the dorsolateral and ventrolateral prefrontal cortices in the manipulation and maintenance of information, respectively (D'Esposito et al., 2000; Petrides, 2005), and the engagement of the frontopolar cortex in the simultaneous operation of cognitive processes (Ramnani and Owen, 2004). Thus, the WM-related activations in these various prefrontal regions may be indicative of a more active operation of these mental processes during performance in the WM load condition.

In contrast, we found that individuals who did not obtain sufficient sleep the night before did not exhibit the same frontal activation pattern, although they performed the *n*-back task as well as did those obtaining sufficient sleep. That is, they exhibited neither significant [oxy-Hb] increases nor [deoxy-Hb] decreases in response to WM load. These neural characteristics are consistent with those reported by most previous fMRI studies that have found frontal underactivation during WM processing following acute total sleep deprivation (Habeck et al., 2004; Choo et al., 2005; Mu et al., 2005; Lythe et al., 2012). However, these studies have also reported impaired WM performance following sleep deprivation, and these findings are not consistent with the present finding. One probable reason for this discrepancy is that, whereas previous studies have focused on total sleep deprivation, the present study focused on partial sleep deprivation. Because the IS group could be characterized as having acute partial sleep deprivation and the effect of partial sleep deprivation on WM appears to be accumulative (Casement et al., 2006; Del Angel et al., 2015), it is speculated that frontal lobe dysfunction may precede evident WM difficulties in the initial neurocognitive consequence of sleep restriction; if the sleep restriction is prolonged (i.e., chronic partial sleep deprivation) or if the sleep deprivation is very severe (i.e., total sleep deprivation), frontal lobe and WM dysfunction might occur at the same time. To verify this speculation, further experimental work that examines the courses of both frontal lobe and WM functioning over consecutive days of partial sleep deprivation and that directly compares total and partial sleep deprivation is needed.

In addition, the intact WM performance despite a lack of WM-related frontal activation in the IS group suggests that other brain regions may compensate for the dysfunctional prefrontal regions during the WM task. Possible neural candidates for such compensation, for instance, are the anterior cingulate and parietal cortices because these regions have been shown to also be activated during WM processing (Owen et al., 2005) and affected by total sleep deprivation (Mu et al., 2005; Lythe et al., 2012). However, because the present NIRS measurement was confined to the prefrontal regions, any changes in activation level in the parietal cortex or other brain regions would have gone undetected. Thus, while the present study has provided evidence for frontal lobe dysfunction during WM performance following acute partial sleep deprivation, further studies that examine neural activity in different parts of the brain (e.g., fMRI studies) are necessary to gain a better understanding of the change of interplay in different brain regions during WM performance associated with (acute) partial sleep deprivation.

Furthermore, it is speculated that several neurophysiological mechanisms may be responsible for the altered frontal processing in the IS group. For example, the WM-related frontal underactivation in the IS group might be partially attributed to metabolic disturbances in frontal brain regions (Wu et al., 2006; Xu et al., 2016). In addition, a partial loss of slow-wave sleep at night may affect synaptic communication in frontal brain regions (Verweij et al., 2014), resulting in altered frontal activation during WM performance on the following day, as was evident in the IS group. Moreover, acute sleep restriction may cause psychological effects that in turn affect brain activation during WM tasks. For example, the acute sleep restriction in the IS group might be associated with an increase in subjective sleepiness level and a reduction in motivation. Thus, participants in the IS group might employ a less effortful strategy when performing the WM task and exhibit a lack of WM-related prefrontal activation.

To our knowledge, one prior study has applied NIRS to investigate the relationship between partial sleep deprivation and frontal lobe processing during cognitive tasks (Miyata et al., 2010). In this study, using a verbal fluency task, the authors found that when participants obtained 8 h of sleep the previous night, their bilateral prefrontal cortices exhibited large activations indicated by large [oxy-Hb] increases during task performance. However, when they obtained less than 4 h of sleep instead, there was a significant reduction in their task-related [oxy-Hb] changes. In addition, it was found that this frontal underactivation as a result of acute partial sleep restriction occurred in the absence of a significant impairment in task performance. Hence, these findings are consistent with the present findings, and our study contributes to the literature by demonstrating that frontal lobe dysfunction may also be evident in the absence of any observable WM difficulties in the initial stage of sleep restriction.

Our findings have several important implications for both the scientific community and the public. First, sleep restriction has been shown to be increasingly common in modern societies (Lund et al., 2010; National Sleep Foundation, 2013; Vargas et al., 2014; Quick et al., 2016). Given that the present study demonstrated the presence of frontal lobe dysfunction in individuals with acute partial sleep deprivation, awareness should be brought to the populations that often experience sleep restriction, such as college students and adults who occasionally have to go to bed late because of overtime work and have to get up early to go to school or work. Second, the present findings provide empirical neuroimaging support for the nightly sleep durations recommended by various sleep organizations, including the National Sleep Foundation, American Academy of Sleep Medicine, and Sleep Research Society (Hirshkowitz et al., 2015; Watson et al., 2015). Third, it should be noted that the present neural activation pattern in individuals with partial sleep deprivation resembles that exhibited in certain neuropathological conditions, such as major depressive disorder (Pu et al., 2011), and schizophrenia (Koike et al., 2013). Given that sleep disturbances are also common in these populations (Cohrs, 2008; van Mill et al., 2010), sleep restriction may foster the development of neuropsychiatric conditions through disturbance in frontal lobe functioning. Nevertheless, further studies are needed to clarify the relationship between sleep deprivation and development of neuropsychiatric symptoms. Finally, the present study provides further empirical support for the use of NIRS to study the neural processing underlying WM performance. Given that NIRS is relatively cost-effective, user-friendly, and environmentally unconstrained, it may be easily applied to research and clinical settings to gain a deeper understanding of the neural processes underlying sleep both in the healthy population and in populations with sleep problems.

Although the present study has enhanced our understanding of the association between partial sleep deprivation and neural processing during WM performance, some limitations need to be mentioned. First, the NIRS measurement was confined to the anterior and lateral prefrontal regions. Given that WM processing may also involve other frontal and non-frontal regions (Owen et al., 2005), participants in the IS group might exhibit altered activation in these regions in compensation for the prefrontal cortices, yet these activation changes might have gone undetected by our measurement. In addition, the IS group represents individuals with mild to moderate acute partial sleep deprivation only (i.e., 4.0–7.0 h the night before). Further studies that include individuals with more severe sleep-deprived conditions (e.g., <4.0 h of sleep) or those with oversleeping conditions (e.g., >10.0 h of sleep) may enhance our understanding of the associations between suboptimal sleep durations and frontal lobe functioning. Furthermore, sleep duration was based on self-reports. Although some studies have reported significant moderate-to-large correlations between self-reported and measured sleep duration (Lauderdale et al., 2008; Cespedes et al., 2016), further studies that utilize objective measurements of sleep duration, such as actigraphy or polysomnography, are needed to verify the present findings.

In addition, while participants in the IS group self-reported obtaining significantly less sleep the night before as compared with their habitual sleep duration, their subjective sleepiness levels were not explicitly measured. Furthermore, caffeine use was not controlled. Thus, further studies that assess caffeine use and measure subjective sleepiness using validated questionnaires may help clarify the mechanisms underlying the link between acute sleep restriction and frontal lobe dysfunction during WM processing as reported in the present study. Specifically, these studies may use NIRS to examine the effects of sleep deprivation, caffeine use, and subjective sleepiness level on WM and WM-related neural processing. Finally, females were over-represented in the sample, and only young adults were included. Thus, further investigations with samples that have a more equal gender distribution and a wider age range are warranted.

In conclusion, the present study utilized NIRS to examine neural processing during WM performance in individuals with and without partial sleep deprivation. We found that unlike individuals who obtained sufficient sleep the night before, those who did not exhibited a lack of bilateral frontal activation during WM processing. This WM-related frontal underactivation was further found to be uniquely associated with the effect of acute partial sleep deprivation, such that individuals who had more severe sleep restriction over the previous night had a greater reduction in the levels of WM-related frontal activation. Hence, the present findings suggest the presence of frontal lobe dysfunction in the absence of evident WM difficulties in individuals with acute partial sleep deprivation. They also highlight the importance of a good night's sleep to brain health.

## Data availability

Data that form the basis of the results reported in the present article are available upon reasonable request.

## Author contributions

MY and AC contributed to the conceptualization of the study. MY and TL contributed to the subject recruitment and data collection. MY undertook the data analysis and prepared the initial draft of the manuscript. MY, TL, WC, and AC significantly contributed to the later versions of the manuscript, read and approved the final version of the manuscript.

### Conflict of interest statement

The authors declare that the research was conducted in the absence of any commercial or financial relationships that could be construed as a potential conflict of interest. The reviewer RZ and handling editor declared their shared affiliation.
